# Rituximab with dose-adjusted EPOCH as first-line treatment in patients with highly aggressive diffuse large B-cell lymphoma and autologous stem cell transplantation in selected patients

**DOI:** 10.3325/cmj.2017.58.40

**Published:** 2017-02

**Authors:** Vlatko Pejša, Željko Prka, Marko Lucijanić, Zdravko Mitrović, Mario Piršić, Ozren Jakšić, Radmila Ajduković, Rajko Kušec

**Affiliations:** 1 Department of Hematology, University Hospital Dubrava, Zagreb, Croatia; 2 School of Medicine, University of Zagreb, Zagreb, Croatia

## Abstract

**Aim:**

To assess the benefit of rituximab with dose-adjusted etoposide, prednisone, vincristine, cyclophosphamide, and doxorubicin (R-DA-EPOCH) regimen as a first-line treatment for patients with diffuse large B-cell lymphoma (DLBCL) presenting with unfavorable or aggressive features, and autologous stem cell transplantation (ASCT) as a part of the first-line treatment for selected DLBCL patients with additional aggressive features.

**Methods:**

We retrospectively analyzed 75 newly diagnosed DLBCL patients with Ki-67+≥80% or International Prognostic Index ≥2 who were treated with R-DA-EPOCH between 2005 and 2015. Of 24 DLBCL patients with additional aggressive features (Ki-67+≥90% or age-adjusted IPI≥2) who were planned to receive consolidation with ASCT, 17 patients underwent the procedure. We determined the overall response rate (ORR), complete remission (CR), partial remission (PR), 5-year overall survival (OS), and progression free survival (PFS) in all DLBCL patients and specifically those planned to receive ASCT.

**Results:**

All 75 patients included in the analysis started one or more cycles of therapy. The ORR, CR, and PR rates were 80%, 55%, and 25%, respectively. The response was non-evaluable in 10 of 75 patients due to treatment discontinuation. The OS and PFS rates for all 75 patients were 70% and 61%, respectively, and 80% and 79%, respectively, for 24 planned-to-receive-ASCT patients. Age (≤65 vs >65 years) had no prognostic impact on OS and PFS (*P* = 0.994 and *P* = 0.827, respectively).

**Conclusion:**

Our retrospective analysis of one of the largest DLBCL patient cohorts outside the US National Cancer Institute showed that R-DA-EPOCH is a very effective therapeutic option as a first-line treatment of DLBCL patients with unfavorable prognostic features irrespective of their age. ASCT provided additional benefit for DLBCL patients with additional aggressive features.

Diffuse large B-cell lymphoma (DLBCL) is the most common aggressive type of B-cell lymphoma (1). It has multiple morphologic variants and heterogeneous molecular background (2). DLBCL treatment failure depends on a variety of factors, including tumor biology, tumor volume, pharmacokinetics, and pharmacogenomics (3-5). Various prognostic scores were developed to differentiate patients with unfavorable prognosis. The International Prognostic Index (IPI) (6,7) and its derivative, the revised IPI (R-IPI) (8), are the primary clinical tools for outcome prediction in the era of R-CHOP, an immunochemotherapy regimen combining rituximab, cyclophosphamide, doxorubicin hydrochloride, vincristine sulfate, and prednisone. The age-adjusted IPI (aaIPI) has also been extensively used in the studies adopting intensive treatment approaches, such as high-dose therapy and stem cell transplantation (9,10). Even though R-CHOP has been a gold standard in DLBCL therapy, there are raising concerns about the efficacy of this regimen, especially in younger patients with poor prognostic factors (11). Several R-CHOP modifications were explored with the intention to improve upfront treatment but did not provide any significantly better results due to greater toxicity (12-15). However, some aggressive alternative regimens improved the outcomes in selected groups of patients, but their applicability has been restricted to younger patients due to high acute and long-term toxicities (16,17).

A dose-adjusted regimen combining etoposide, prednisone, vincristine, cyclophosphamide, and doxorubicin (DA-EPOCH) was developed as a continuous infusion regimen relying on a concept that tumor cells display relatively less resistance to a prolonged drug exposure in comparison with brief higher concentration exposure (18). Dynamic dose adjustments according to patient’s bone marrow status (observed as the absolute neutrophil and platelet counts) allow for the use of the highest acceptable doses of drugs, while avoiding additional toxicity and improving the results (19). Thus, this treatment approach is suitable for older patients. Rituximab with DA-EPOCH (R-DA-EPOCH) demonstrated efficacy in treatment of patients with DLBCL (20-22). Superior progression-free survival has been observed in a subset of DLBCL patients harboring c-myc and bcl-2 rearrangements, termed “double hit” lymphomas, treated with R-DA-EPOCH regimen in comparison to R-CHOP regimen-treated patients (23,24). The role of autologous stem cell transplantation (ASCT) as part of a first-line therapy in DLBCL is still unresolved. Although it is generally not recommended because it has no impact on the overall survival (25), it might have a role in first-line treatment of patients with poor prognostic features (11,26,27).

The aim of our study was to determine the benefit of R-DA-EPOCH regimen as a first-line treatment for DLBCL patients presenting with unfavorable or aggressive features and ASCT as part of first-line treatment in a subgroup of DLBCL patients with additional aggressive features.

## PATIENTS AND METHODS

This retrospective cohort study was performed at the Department of Hematology, University Hospital Dubrava in Zagreb, Croatia, from May 2005 to December 2015.

### Patients

A total of 75 patients with newly diagnosed DLBCL and unfavorable prognostic features, defined as a very high proliferative index Ki-67+≥80% and/or IPI≥2, were treated with R-DA-EPOCH regimen during the study period (Table 1). A subset of 24 DLBCL patients who initially presented with additional aggressive features (Ki-67+≥90% and/or aaIPI≥2) and had no transplant-limiting comorbidities were planned to undergo ASCT as part of first-line therapy. Seven patients received concomitant radiation therapy. All patients provided written informed consent for therapy.

**Table 1:** Characteristics, treatment, and clinical outcomes in all patients with diffuse large B cell lymphoma (DLBCL) and a subgroup of DLBCL patients planned to receive autologous stem cell transplantation (ASCT)*

	No. of DLBCL patients	
**Characteristics**	**total (n = 75)**	**planned to receive ASCT (n = 24)**
Sex (male/female)	42/33	12/12
Age (years; median, IQR)	61 (47-71)	50 (41-57)
Ann Arbor III & IV	61	21
High LDH	57	21
ECOG≥2	26	11
>1 extranodal presentation	37	12
Bulky disease	28	11
Ki-67 ≥ 80%	31	8
Ki-67 ≥ 90%	18	7
B symptoms	37	15
IPI≥2	64	20
Age-adjusted IPI≥2	-	18
R-IPI poor risk	45	12
R-DA-EPOCH cycles (median, IQR)	6 (6-8)	7 (6-7)
ASCT transplantation	17	17
CD34+ cells mobilized with EPOCH	-	18/21†
Irradiation	7	2

*Abbreviations: IQR – interquartile range; LDH – lactate dehydrogenase; ECOG – Eastern Cooperative Oncology Group; B symptoms – fever, weight loss, and night sweats; IPI –International Prognostic Index; R-IPI – revised IPI; R-DA-EPOCH – rituximab plus dose-adjusted regimen combining etoposide, prednisone, vincristine, cyclophosphamide, and doxorubicin.

^†^Three patients planned to receive ASCT died prior to stem cell mobilization.

#### Chemotherapeutic regimen

Original DA-EPOCH regimen (20) consists of continuous intravenous infusion of etoposide 50 mg/m^2^/d on days 1 to 4, oral prednisone 60 mg/m^2^/d on days 1 to 5, continuous infusion of vincristine (Oncovine) 0.4 mg/m^2^/d on days 1 to 4, cyclophosphamide 750 mg/m^2^/d administered intravenously over 15 minutes on day 5, continuous infusion of doxorubicin (Hydroxydaunorubicin) 10 mg/m^2^/d on days 1 to 4, and filgrastim 5 μg/kg administered subcutaneously from day 6 until the absolute neutrophil count (ANC) reaches 10 × 10^9^/L. The regimen is repeated every three weeks. DA-EPOCH regimen was administered according to original schedule with only one difference that pegylated filgrastim was administered instead of daily filgrastim on day 6 of regimen since it became available in Croatia. All patients had peripheral central line (PICC) inserted before the therapy.

Response evaluation was performed after four cycles of therapy. In case of complete remission (CR), patients received additional two cycles. Otherwise, they received additional four cycles, ie, a total of eight cycles, with the final evaluation performed after the treatment completion. All patients received six to eight cycles of rituximab concomitantly. All patients received *Pneumocystis jiroveci* pneumonia prophylaxis with trimethoprim-sulfamethoxazole twice a day, three times a week. Some patients also received antiviral and antifungal prophylaxis with acyclovir and fluconazole at physician’s discretion.

#### ASCT treatment

In patients undergoing ASCT, the last cycle of DA-EPOCH was used as a stem cell mobilization regimen. In these settings, filgrastim administration was postponed after ANC nadir was reached, approximately 7-10 days after the last cycle of therapy. If unsuccessful, mini-BEAM (carmustine, etoposide, cytarabine, melphalan) chemotherapy or plerixafor were used before the stem cell apheresis. BEAM regimen was used for myeloablation.

### Method

All patients that started one or more cycles of therapy or were planned to receive ASCT as a part of a first line therapy were included into analyses, thereby mimicking intention to treat approach. Response to therapy was reported as overall response rate (ORR), complete remission (CR), partial remission (PR), progressive disease, and non-evaluable if treatment had to be discontinued before the disease evaluation could be performed due to reasons other than disease progression. Survival endpoints including death for overall survival (OS) and death, progression, and relapse for progression free survival (PFS) were calculated from the first day of therapy to the day of death, disease progression, relapse, or last follow-up as appropriate. Patients who discontinued R-DA-EPOCH due to toxicity and continued treatment with alternative regimen were censored at the time of treatment discontinuation. Patients who discontinued R-DA-EPOCH due to disease progression or died during the treatment were marked as achieving an endpoint.

### Statistical analysis

Normality of distribution of numerical variables was tested using the Kolmogorov-Smirnov test. Since most numerical variables were non-normally distributed, they are presented as medians with interquartile range (IQR). Categorical variables were presented as proportions. Survival analyses were performed using Kaplan-Meier method (28) and the log-rank test (29). The median follow-up was estimated using reverse Kaplan-Meier estimator (30). Data were screened using custom made MS Excel workbook (31) and reanalyzed using MedCalc Statistical Software version 16.2.0 (MedCalc Software bvba, Ostend, Belgium). *P* < 0.05 was considered statistically significant.

## RESULTS

### Clinical outcomes

The response to therapy was achieved in 60 of 75 treated patients (ORR 80%). Of 75 treated patients, 41 (55%) achieved CR, 19 (25%) achieved PR, and 5 (7%) had progressive disease. The response was non-evaluable in 10 of 75 patients due to treatment discontinuation. When analyzed in evaluable patients only, response rates were 60/65 (92%), 41/65 (63%), and 5/65 (29%) for ORR, CR, and PR, respectively.

**Figure 1:**
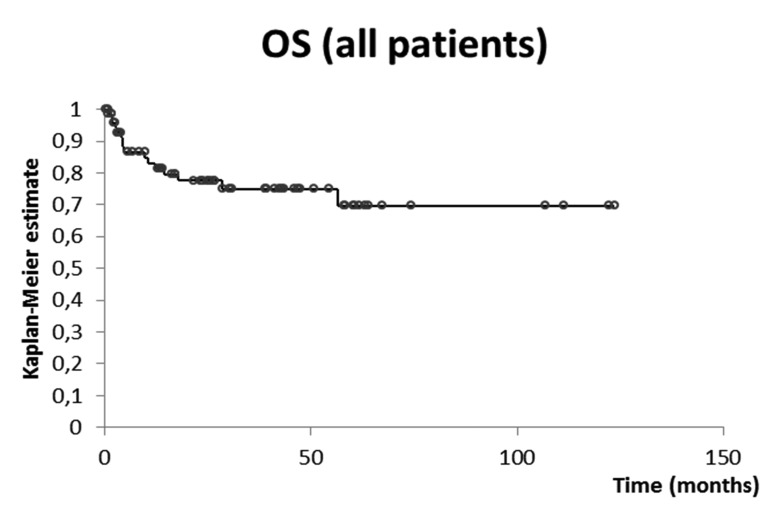
Overall survival (OS) in all 75 patients with diffuse large B-cell lymphoma. The 5-year and 3-year survival rates were 75% and 70%, respectively.

**Figure 2:**
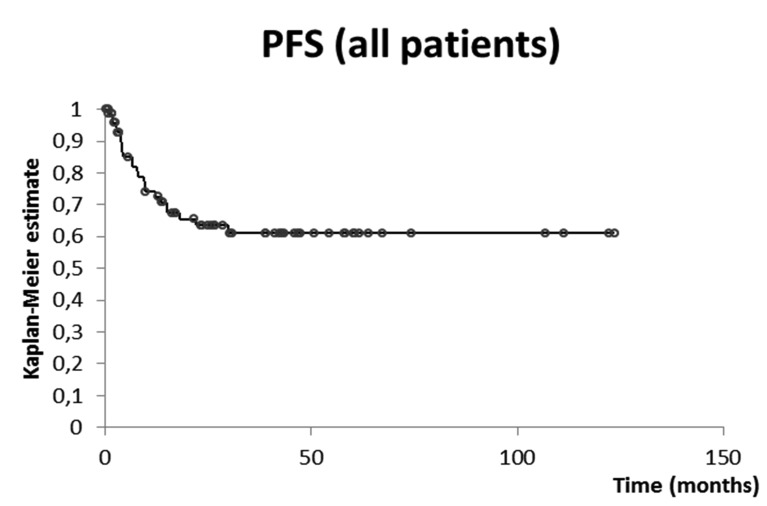
Progression free survival (PFS) in all 75 patients with diffuse large B-cell lymphoma. The 5-year and 3-year survival rates were both 61%.

**Figure 3:**
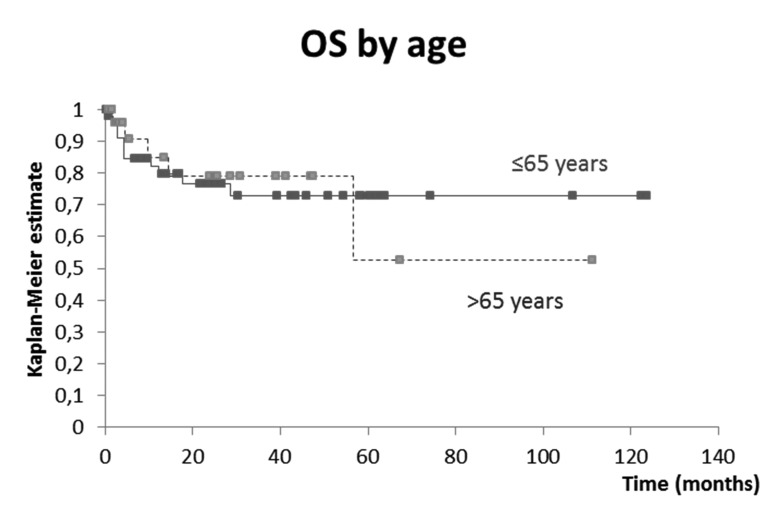
Age impact on overall survival (OS) in all 75 patients with diffuse large B-cell lymphoma. There was no difference in OS between patients aged ≤65 years (full line) and >65 years (dashed line), *P* = 0.994.

**Figure 4:**
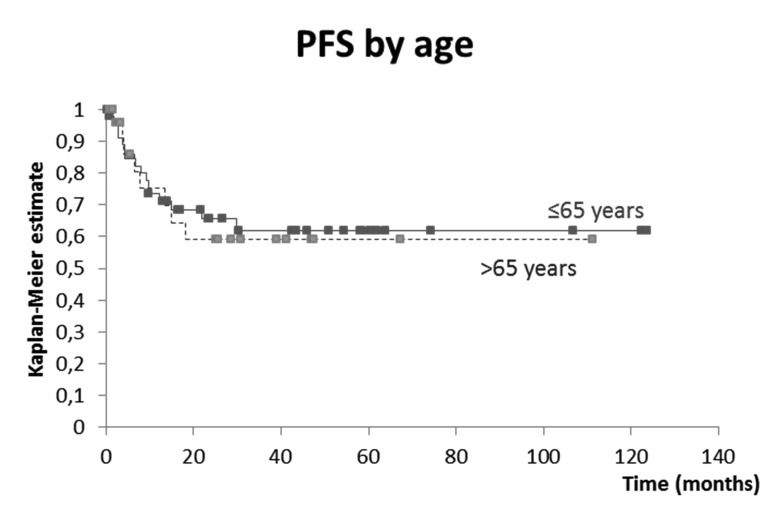
Progression free survival (PFS) in all 75 patients with diffuse large B-cell lymphoma. There was no difference in PFS between patients aged ≤65 years (full line) and >65 years (dashed line), *P* = 0.827.

The 3-year and 5-year OS rates for all DLBCL patients were 75% and 70%, respectively. The median follow-up time was 29 months, and the median survival time was not reached (Figure 1). The 3-year and 5-year PFS rates for all DLBCL patients were both 61%. The median follow-up time was 39 months, and the median survival time was not reached (Figure 2). As expected, patients achieving CR had superior OS than patients achieving PR (HR 0.11; *P* = 0.001) or not achieving remission at all (HR 0.02; *P* < 0.001). Age (65-year cut-off) had no prognostic impact on OS (Figure 3) or PFS (Figure 4). We did not observe any significant effect of other IPI-contained risk factors (the Eastern Cooperative Oncology Group [ECOG] performance status ≥2, involvement of more than one extranodal site, increased lactate dehydrogenase [LDH], and advanced Ann Arbor stage [≥3]) on OS or PFS. Accordingly, neither IPI nor R-IPI had a prognostic significance for OS or PFS in our cohort of patients. R-IPI-defined poor-risk group of patients showed very good survival rates: 4-year OS and PFS rates with 95% confidence intervals were 78% (64%-91%) and 61% (45%-77%), respectively.

### Feasibility

The 75 patients included in the study received a total of 455 cycles of therapy. The median number of administered cycles was six per patient (IQR 6-8). Dose adjustment was assessed in 71 patients with available data who received a total of 428 cycles. Doses were escalated in 134/428 (31%) cycles, lowered in 27/428 (6%) cycles, and unchanged in 196/428 (46%) cycles, whereas 71/428 (17%) first cycles represent the difference to 100%. The median number of cycles with dose escalation was two per patient (IQR 0-3) and reaching up to 249% of the initial dose in individual patients (dose escalated 4 times).

### Hematological and non-hematological toxicities

Hematological toxicity during the treatment was assessed in 63 patients with available data who received a total of 371 cycles (Table 2). Hematologic toxicity grade 3 or 4 according to National Cancer Institute Common Terminology Criteria for Adverse Events (NCI CTCAE), version 4.0 (32), was detected in all analyzed patients due to toxicity-tailored nature of the regimen itself (dose adjustment according to ANC). All patients with febrile neutropenia were hospitalized and treated accordingly. The median number of cycles in patients with febrile neutropenia was one per patient (IQR 0-2). The median cycle of febrile neutropenia occurrence was cycle four (IQR 2-5).

**Table 2:** Treatment-related hematological and non-hematological toxicities in patients with diffuse large B cell lymphoma

	No. (%) of cycles	No. (%) of patients
Hematological toxicities*		
anemia (grade 3 or 4)	65/371 (18.0)	36/63 (57.0)
thrombocytopenia (grade 3 or 4)	63/371 (17.0)	35/63 (56.0)
neutropenia (grade 4)	152/371 (41.0)	58/63 (92.0)
febrile neutropenia	66/371 (18.0)	40/63 (63.0)
**Non-hematological toxicities**		
venous thrombosis	-	9/75 (12.0)
catheter-related	-	7/75 (9.0)
cerebrovascular infarction	-	1/75 (1.0)
polyneuropathy gr. ≥2	-	11/75 (15.0)
gastrointestinal hemorrhage	-	3/75 (4.0)
hepatitis B reactivation	-	1/75 (1.0)
hemorrhagic cystitis	-	1/75 (1.0)
Treatment discontinuation	-	9/75 (12.0)
Deaths during treatment	-	9/75 (12.0)

*Data available for 63 patients who received a total of 371 cycles.

R-DA-EPOCH had to be discontinued in 9 of 75 patients due to inability to comply with regimen-related logistic requirements (six-day hospitalization, frequent blood sampling between cycles, PICC hygiene), treatment-related toxicity or unsatisfactory disease response to therapy. Nine of 75 patients died during the treatment. Three patients died due to disease progression and six died due to toxicities including fatal sepsis originating in the respiratory tract (n = 2) or skin (n = 2; one catheter-related), fatal hepatitis B reactivation (n = 1), and thrombocytopenia-associated fatal gastrointestinal hemorrhage (n = 1). We observed no significant doxorubicin-related cardiac toxicities. No secondary malignancies were detected.

### ASCT subcohort

**Figure 5:**
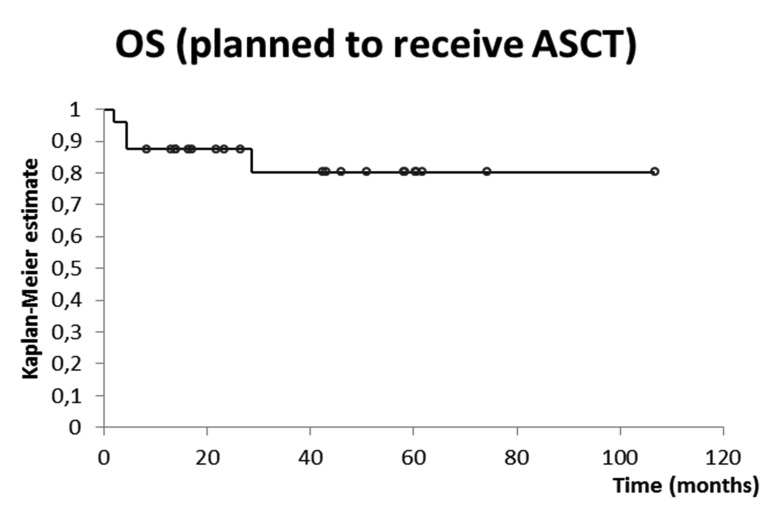
Overall survival (OS) in 24 patients with diffuse large B-cell lymphoma planned to receive autologous stem cell transplantation (ASCT).

**Figure 6:**
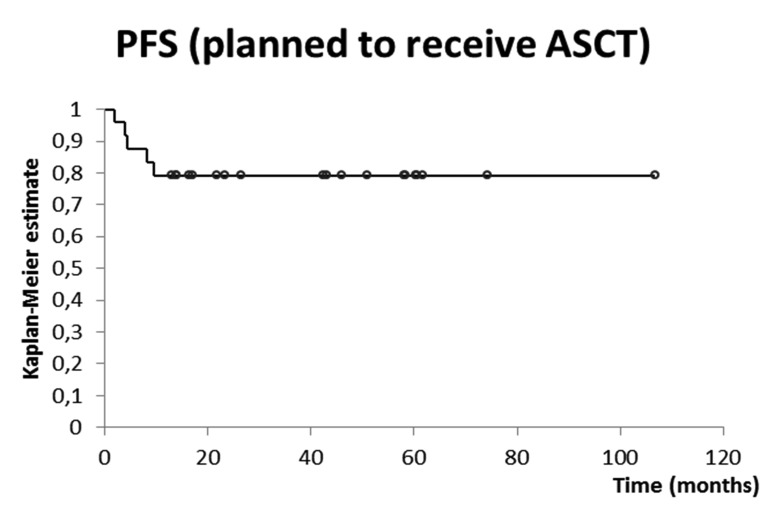
Progression free survival (PFS) in 24 patients with diffuse large B-cell lymphoma planned to receive autologous stem cell transplantation (ASCT).

In the subset of 24 patients planed to receive ASCT, ORR was achieved in 21, CR in 15, and PR in 6 patients, whereas the response was not evaluable in 2 patients. The 3-year and 5-year OS rates were 80%. The median follow-up time was 43 months, whereas the median survival time was not reached (Figure 5). The 3-year and 5-year PFS rates in these 24 patients were both 79%. The median follow-up time was 43 months, and the median survival time was not reached (Figure 6).

Of 24 patients who were planned to receive ASCT, three died during the R-DA-EPOCH treatment (two due to infective complications and one due to disease progression). In 21 patients who completed R-DA-EPOCH, the last cycle of DA-EPOCH was used as a stem cell mobilizing regimen with 86% success rate (18 of 21 patients). The median time from the start of last cycle to the first stem cell apheresis was 17 days (IQR 16-18). Seventeen of 21 patients underwent ASCT procedure, one patient withdrew the consent for the procedure, and three patients failed to collect adequate graft. During ASCT, 13 of 17 patients developed febrile neutropenia and one patient developed neutropenic colitis requiring surgical intervention. The median time to ANC recovery was 11 days (IQR 10-11), and the median time to platelet count recovery was 12 days (IQR 12–14). There were no transplantation-related deaths, additional cardiac toxicities or secondary malignancies observed in this group of patients.

## DISCUSSION

We found that R-DA-EPOCH followed by ASCT is an effective first-line treatment approach in DLBCL patients with unfavorable prognostic features. R-DA-EPOCH can surpass a negative prognostic impact of age on survival of patients. Our results also suggest that R-DA-EPOCH offers improved survival benefit in R-IPI defined “poor-risk” patients in comparison with previously reported 4-year OS rate of 55% in R-CHOP-treated patients (8).

DA-EPOCH was initially developed and used as the first-line treatment for DLBCL patients at the US National Cancer Institute, with the reported 5-year OS and PFS rates of 73% and 70%, respectively (20). In 2007, a Spanish group reported using R-DA-EPOCH for the treatment of poor prognosis DLBCL patients and achieving 2-year OS and event-free survival (EFS) rates of 75% and 68%, respectively, with aaIPI≥3 as the only factor related to poor EFS (21). Recently, another group from Spain reported 10-year OS and EFS rates of 64% and 48%, respectively, in R-DA-EPOCH-treated patients with poor prognosis DLBCL (33). ASCT has been extensively discussed as part of first-line therapy for aggressive non-Hodgkin lymphomas, especially DLBCL (26,34-36), where consolidation using high-dose therapy and ASCT was shown to reduce relapse rate, mainly in a subgroup of high-risk patients (37-39). In 2008, Greb et al (25) reported a Cochrane database review including more than 3000 patients and concluded that initial CR rate superiority did not translate into OS, ie, OS and EFS were the same regardless of whether the patients were treated with conventional chemotherapy or high-dose therapy followed by ASCT. This lack of survival benefit was reason not to include or recommend ASCT as a standard part of first-line therapy. Additional data from the results of the SWOG Intergroup phase III trial demonstrated that the addition of ASCT resulted in a significantly higher 2-year PFS but no difference in 2-year OS was observed. Subset analyses showed that the benefit of ASCT was found primarily in patients with high-IPI disease, in whom 2-year OS rate was 82% (ASCT) vs 64% (27). Our results regarding the 5-year OS and PFS in our DLBCL patients and those planned to receive ASCT are in line with previous experiences with R-DA-EPOCH regimen in similar cohorts of patients. As suggested by PFS survival curves, our patients achieving remission and not progressing over 2 years of follow-up are probably cured.

Our study sample included some patients with IPI<2 but with a very high proliferation index (and otherwise unfavorable disease features), which is partly the reason why IPI and R-IPI showed no prognostic significance. Also, dynamic dose adjustment tailored to the hematopoietic reserve of individual patients allowed for the application of the highest acceptable doses and maximization of therapeutic effect. This approach inevitably results in an increased hematologic toxicity, but it enables optimal treatment of elderly patients and abrogates negative prognostic significance of age on survival (included into IPI and R-IPI scores). Increased hematologic toxicity, treatment discontinuation, and deaths during treatment do not offset high survival rates in our cohort of patients. Further benefit observed was the ability to use the last cycle of DA-EPOCH therapy for stem cell mobilization with 86% success rate, reducing the need for additional stem cell mobilizing regimen and associated risks.

Experience with combination of DA-EPOCH with ASCT as part of first-line therapy for poor-risk non-Hodgkin lymphomas is still lacking despite the promising results with double-hit lymphomas (40). Although we were unable to discriminate patients with double-hit lymphomas, our inclusion criteria based on very high proliferation rates and unfavorable prognostic features probably pooled such patients. Results of immunochemotherapy regimens in double-hit lymphomas are unsatisfactory. Petrich et al (40) reported median PFS and OS rates for the entire cohort of 10.9 and 21.9 months, respectively, and the PFS and OS rates at 2 years of 40% and 49%, respectively. In the first meta-analysis of patients with double-hit lymphomas, Howlett et al (23) found that median PFS for the R-CHOP, R-EPOCH, and other intensive regimens groups was 12.1, 22.2, and 18.9 months, respectively. First-line treatment with R-EPOCH significantly reduced the relative risk of a progression compared with R-CHOP by 34%; however, OS was not significantly different across treatment approaches. They also presumed that a subset of patients might benefit from intensive induction with or without transplant (23). These results showed that R-DA-EPOCH is the only regimen that provides PFS advantage, but without OS benefit. Most patients with double-hit lymphomas are within the group of DLBCL patients with high Ki-67%. Their treatment results are significantly worse than in other DLBCL patients and those in our cohort. Thus, we assume that patients with double-hit lymphomas comprised only a minority among our patients.

In search for better treatment options for high-risk DLBCL patients, many intensive regimens were evaluated with mostly dose densing or intensification and etoposide addition. Schmitz et al (15) reported the 3-year EFS of 69.5% and 61.4% in the R-CHOEP14- and R-MegaCHOEP-treated patients, respectively, without a statistical significance. Doubling and densing rituximab therapy in addition to CHOEP14 did not improve results (41). However, these were all young patients with high-risk disease. The results of R-CHOP treatment in older patients are not so favorable. In the International Society of Geriatric Oncology (SIOG) expert position commentary by Morrison et al (42), CR rates were 50% in patients aged 65–75 years, and 40% in those aged >75 years. Median remission duration was 16 months; cure rates were 50%–60% in younger and 25%–30% in older patients.

Our study had a few limitations, including retrospective design, single-center experience, and inability to discriminate double-hit lymphomas from other DLBCL subsets. High logistic requirements of R-DA-EPOCH regimen resulted in lower threshold for treatment discontinuation in some patients experiencing otherwise manageable complications. The effects of treatment discontinuation on outcome measures were taken into account by analyzing the treatment response in both the complete cohort and evaluable patients only, and by using strict inclusion and censoring criteria regarding the patients who progressed or died during treatment (we included all patients who had received ≥1 cycle of therapy). The strengths of our study are mimicking the intention-to-treat approach, respectable number of patients treated, and long follow-up.

In conclusion, our results in one of the largest cohorts of patients outside the US National Cancer Institute showed that R-DA-EPOCH is a very effective therapeutic option as the first-line treatment for DLBCL patients with unfavorable prognostic features irrespective of their age. ASCT provides additional benefit for a selected group of patients with acceptable toxicity.
